# Synthesis and in vitro assay of hydroxyxanthones as antioxidant and anticancer agents

**DOI:** 10.1038/s41598-022-05573-5

**Published:** 2022-01-27

**Authors:** Nela Fatmasari, Yehezkiel Steven Kurniawan, Jumina Jumina, Chairil Anwar, Yoga Priastomo, Harno Dwi Pranowo, Abdul Karim Zulkarnain, Eti Nurwening Sholikhah

**Affiliations:** 1 Department of Chemistry, Faculty of Mathematics and Natural Science, Universitas Gadjah Mada, Yogyakarta, 55281 Indonesia; 2 Department of Pharmaceutical Technology, Faculty of Pharmacy, Universitas Gadjah Mada, Yogyakarta, 55281 Indonesia; 3 Department of Pharmacology and Therapy, Faculty of Medicine, Public Health, and Nursing, Universitas Gadjah Mada, Yogyakarta, 55281 Indonesia

**Keywords:** Cancer, Drug discovery, Molecular medicine, Chemistry

## Abstract

In the present work, three hydroxyxanthones were synthesized in 11.15–33.42% yield from 2,6-dihydroxybenzoic acid as the starting material. The chemical structures of prepared hydroxyxanthones have been elucidated by using spectroscopic techniques. Afterward, the hydroxyxanthones were evaluated as antioxidant agents through radical scavenging assay; and anticancer agents through in vitro assays against WiDr, MCF-7, and HeLa cancer cell lines. Hydroxyxanthone **3b** was categorized as a strong antioxidant agent (IC_50_ = 349 ± 68 µM), while the other compounds were categorized as moderate antioxidant agents (IC_50_ > 500 µM). On the other hand, hydroxyxanthone **3a** exhibited the highest anticancer activity (IC_50_ = 184 ± 15 µM) and the highest selectivity (SI = 18.42) against MCF-7 cancer cells. From the molecular docking study, it was found that hydroxyxanthone **3a** interacted with the active sites of Topoisomerase II protein through Hydrogen bonding with DG13 and π–π stacking interactions with DA12 and DC8. These findings revealed that hydroxyxanthones are potential candidates to be developed as antioxidant and anticancer agents in the future.

## Introduction

Oxidative stress diseases have been receiving great attention due to their harmful, severe, and unfavorable effects on the human body^1^. Several fatal diseases such as cancer, arthritis, cardiovascular, and neurodegenerative diseases are generated by oxidative stress that damages human tissue and organs^2^. Free radicals are reported as the main factor in the production of oxidative stress in the human body. Free radicals are very reactive; thus, they react rapidly with biomolecules such as DNA and affect cell metabolism^3^. Additionally, free radicals are also responsible for cholesterol and atherosclerosis deposition inside the blood vessels^4^. Therefore, researchers are making extensive efforts to prevent and deactivate the free radicals before damaging the cell function^5^.

Usage of antioxidant agents is the most straightforward approach to deactivate the free radicals^6^. Almost all antioxidant agents are composed of phenolic compounds because phenolic compounds are able to generate hydrogen donor and electron delocalization mechanisms to suppress the formation of oxidative stress^7^. Several natural and synthetic antioxidant agents such as chalcone, indole-3-acetamide, cadmium-bismuth microsphere, indazole, and salicylhydrazidehydrazone derivatives have been evaluated; however, their chemical synthesis is not easy, and their antioxidant activity is still unsatisfactory^8–12^.

Almost 80–95% of antioxidant and anticancer agents consist of a heterocyclic structure. Among the heterocyclic compounds, xanthone derivatives with a dibenzo-γ-pyrone skeleton exhibit unique physicochemical properties^13–16^. Furthermore, xanthones display broad pharmacological activities, which are remarkable to be further developed for the drug research^17–21^. Xanthone could be obtained from various plants, such as *Symphonia globulifera* and *Garcinia mangostana*^22,23^. Nevertheless, the isolation process is very complicated to obtain the desired xanthone derivatives in high purity. Furthermore, the isolation and purification of xanthones require a large volume of organic solvents in a time-consuming process^24^. Therefore, the synthesis of xanthone is much attractive to be developed to afford xanthone derivatives in a higher yield within a faster research period. Moreover, it is possible to obtain xanthone derivatives with specific functional groups at a certain position by using a synthesis reaction, which may not be found in natural sources^18^.

The synthesis of xanthone was firstly established in 1892 by Michael and Kostanecki by reacting phenol, acetic acid-*o*-hydroxybenzoate, and acetic anhydride. Unfortunately, this method generates an unfavorable side reaction; thus, the obtained yield was very low. Eaton’s reagent (a mixture of P_2_O_5_ and CH_3_SO_3_H) has recently been introduced to give a higher yield for xanthone derivatives^25^. For example, Zhou et al. successfully synthesized 1,3-dihydroxydinitroxanthone and 7-(2,4-diphenyl)-1,3-dihydroxyxanthone in up to 76% yield; however, their antioxidant activity was still lower compared to the commercial antioxidant agents^26^. Because of that, synthesis and modification of other xanthone derivatives are important to find the active antioxidant agents to be used for commercial purposes.

Xanthone, benzylhydrazone, and quinoxaline derivatives have been reported to give remarkable anticancer activities^16,27,28^. Hydroxyxanthones exhibit good anticancer activity against lung, breast, hepatoma, cervical, colorectal, and other cancer cell lines^16,29^. Hydroxyxanthones could lead to cell apoptosis by stimulating the caspase enzyme^30^. Furthermore, hydroxyxanthones are able to inhibit Topoisomerase II protein by intercalation mechanism on the DNA cleavage sites^31^. This anticancer mechanism occurred as the hydroxyxanthones were constructed by three fused aromatic systems, which were reported as the essential pharmacophoric feature of Topoisomerase II inhibitors^32^. Furthermore, the three fused aromatic structure of hydroxyxanthones is similar to doxorubicin and mitoxantrone as standard DNA intercalators^28^. Therefore, hydroxyxanthones are potential to be further investigated as anticancer agents.

In our previous work, several hydroxyxanthone derivatives, i.e., 1,3-dihydroxyxanthone, 3,4-dihydroxyxanthone, 3,6-dihydroxyxanthone, 1,3,6-trihydroxyxanthone, and 3,4,6-trihydroxyxanthone have been synthesized and evaluated as the anticancer agent against WiDr cancer cell line^33^. In continuation of our previous research, we synthesized other hydroxyxanthone derivatives to give a broader map of their antioxidant and anticancer activities through in vitro and molecular docking studies. In this work, three hydroxyxanthone derivatives, i.e., 1,3,8-trihydroxyxanthone (**3a**), 1,6-dihydroxyxanthone (**3b**), and 1,5,6-trihydrokxyxanthone (**3c**) were prepared from 2,6-dihydroxybenzoic acid with phloroglucinol, resorcinol, and pyrogallol, respectively. The chemical structure of these hydroxyxanthones was elucidated using Fourier transform infrared (FTIR) spectrophotometer, mass spectrometry (MS), and nuclear magnetic resonance (NMR) spectrometers. These compounds were evaluated as antioxidant and anticancer agents through in vitro assay using 1,1-diphenylpicryl-2-hydrazyl (DPPH) and 3-(4,5-dimethylthiazol-2-yl)-2,5-diphenyltetrazolium bromide (MTT) methods, respectively. The inhibition mechanism of the most active anticancer agent against Topoisomerase II protein was further studied through a molecular docking study.

## Materials and methods

### Materials

The chemicals used in the synthesis, i.e., 2,6-dihydroxybenzoic acid (C_7_H_6_O_4_), phloroglucinol (C_6_H_6_O_3_), resorcinol (C_6_H_6_O_2_), pyrogallol (C_6_H_6_O_3_), Eaton’s reagent (P_2_O_5_, CH_3_SO_3_H), DPPH (C_18_H_12_N_5_O_6_), *n*-hexane (C_6_H_14_), ethyl acetate (C_4_H_8_O_2_), and methanol (CH_3_OH) were purchased from Merck in pro analytical grade. In addition, butylated hydroxytoluene (BHT, C_15_H_24_O) and MTT (C_18_H_17_N_5_S) were obtained from Aldrich in pro analytical grade.

### Preparation of hydroxyxanthones

A mixture of 2,4-dihydroxybenzoic acid (3.66 g, 15.0 mmol), the substituted phenol (phloroglucinol, resorcinol, and pyrogallol) (15.0 mmol, 1 equiv.), and Eaton’s reagent (8.00 mL) was heated at 80–85 °C for 3 h. Then, the mixture was allowed to reach room temperature, and the mixture was poured with cooled water (50 mL). The resulting solid residue was collected by filtration, washed with water until neutral, dried, and then purified by preparative thin layer chromatography. In the preparative thin-layer chromatography, silica was used as the stationary phase, while a mixture of *n*-hexane and ethyl acetate 1:1 v/v was used as the mobile phase to afford the target compound. The products were then characterized using FTIR (Shimadzu-Prestige 21), MS (Shimadzu QP-2010S), ^1^H– and ^13^C–NMR (JEOL JNMECA 500 MHz) spectrometers.

1,3,8-trihydroxyxanthones (**3a**). The compound **3a** was obtained as a yellow solid in 16.14% yield. m.p. 239–240 °C. FTIR (KBr) v/cm^–1^: 3448 (O–H), 1612 (C = O), 1418 (C = C), 1296 (C–O–C). ^1^H–NMR (CD_3_OD, 500 MHz) δ/ppm = 6.09 (1H, d, *J* = 2.07 Hz, H7), 6.20 (1H, d, *J* = 2.07 Hz, H5), 6.64 (1H, d, *J* = 8.50 Hz, H2), 6.78 (1H, d, *J* = 8.50 Hz, H4), 7.49 (1H, t, *J* = 8.36 Hz, H3). ^13^C–NMR (CD_3_OD, 125 MHz) δ/ppm = 95.8 (C7), 100.0 (C5), 107.9 (C8a), 111.4 (C8b), 115.9 (C4), 128.4 (C2), 137.6 (C3), 157.4 (C4a), 159.4 (C4b), 162.3 (C1), 164.2 (C6), 169.9 (C8), 185.1 (C9). MS *m/z* = 244 [M]^+^.

1,6-dihydroxyxanthones (**3b**). This compound was obtained as a yellow solid in 33.42% yield; m.p. 248–249 °C. FTIR (KBr) v/cm^–1^: 3425 (O–H), 1604 (C = O), 1465 (C = C), 1273 (C–O–C). ^1^H–NMR (CD_3_OD, 500 MHz) δ/ppm = 6.67 (1H, dd, *J* = 2.15 and 8.81 Hz, H7), 6.71 (1H, d, *J* = 2.15 Hz, H5), 6.81 (1H, dd, *J* = 8.31 and 2.23 Hz, H2), 6.85 (1H, dd, *J* = 8.31 and 2.23 Hz, H4), 7.52 (1H, t, *J* = 8.31 Hz, H3), 7.99 (1H, d, *J* = 8.81 Hz, H8). ^13^C–NMR (CD_3_OD, 125 MHz) δ/ppm = 102.7 (C5), 107.3 (C4), 108.8 (C8b), 110.5 (C2), 113.2 (C8a), 115.4 (C7), 127.9 (C8), 136.6 (C3), 157.2 (C4a), 159.4 (C4b), 162.4 (C6), 167.5 (C1), 181.9 (C9). MS *m/z* = 228 [M]^+^.

1,5,6-trihydroxyxanthones (**3c**). This compound was obtained as a yellow solid in 11.15% yield. m.p. 251–252 °C. FTIR (KBr) v/cm^–1^: 3448 (O–H), 1604 (C = O), 1458 (C = C), 1273 (C–O–C). ^1^H–NMR (CD_3_OD, 500 MHz) δ/ppm = 6.67 (1H, dd, *J* = 8.31 and 2.23 Hz, H4), 6.78 (1H, dd, *J* = 8.31 and 2.23 Hz, H2), 6.84 (1H, d, *J* = 8.94 Hz, H7), 7.51 (1H, t, *J* = 8.31 Hz, H3), 7.97 (1H, d, *J* = 8.94 Hz, H8). ^13^C–NMR (CD_3_OD, 125 MHz) δ/ppm = 103.4 (C4), 107.8 (C8b), 109.4 (C2), 110.9 (C7), 113.2 (C8a), 116.6 (C8), 128.3 (C5), 137.0 (C3), 157.7 (C4b), 160.1 (C6), 162.9 (C4a), 169.4 (C1), 182.3 (C9). MS *m/z* = 228 [M–16]^+^.

### In vitro antioxidant assay

The antioxidant activity of hydroxyxanthones was evaluated by using a DPPH scavenging assay. Each hydroxyxanthone (**3a**-**c**) was dissolved in methanol at a concentration range from 10 to 100 µg mL^–1^. The solution was then mixed with 100 µg mL^–1^ DPPH in methanol and allowed for 30 min in a dark condition. The absorbance value of the absorption signal at 517 nm was measured using a UV–Vis spectrophotometer (Shimadzu UV 1800). The radical scavenging percentage was calculated using Eq. (1), whereas A_blank_ and A_sample_ are the absorbances at 517 nm for blank (methanol) and sample (hydroxyxanthone), respectively.

1 $$ \% {\text{ Radical scavenging }} = \, \left( {{\text{A}}_{{{\text{blank}}}} {-}{\text{A}}_{{{\text{sample}}}} } \right)/\left( {{\text{A}}_{{{\text{blank}}}} } \right) \, \times { 1}00\% $$

Afterward, the radical scavenging percentage was plotted versus the concentration of the antioxidant agent. The half-maximal inhibitory concentration (IC_50_) value was calculated by fixing the radical scavenging percentage equal to 50%. The in vitro antioxidant assay for each sample was performed in three replications.

### In vitro anticancer assay

The in vitro anticancer activity of hydroxyxanthones was evaluated by using the MTT method. All cancer and normal (Vero) cell lines were supplied by the Parasitology Laboratory, Universitas Gadjah Mada, Indonesia. The cells were cultured at 37 °C in an incubator containing 5% CO_2_. Briefly, the WiDr (colorectal cancer), MCF-7 (breast cancer), and HeLa (cervical cancer) cells were suspended in medium with 10% Fetal Bovine Serum. Concisely, the cell lines were added to 96-well plates and cultured in a medium with various concentrations of each hydroxyxanthone **3a**–**c**. The mixture was stored in a CO_2_ incubator. After 24 h, the MTT reagent was added, and the mixture was further incubated for an additional 3 h. Afterward, the sodium dodecyl sulfate (SDS) page was added to the mixture, and the mixture was re-incubated for 24 h in a darkroom. The absorbance value of the solution at 495 nm was measured using an Elisa reader (Benchmark) to calculate viability cells’ percentage (Eq. 2).

2 $$ \% {\text{Viability cells }} = \, ({\text{A}}_{{{\text{blank}}}} {-}{\text{A}}_{{{\text{sample}})}} /\left( {{\text{A}}_{{{\text{blank}}}} } \right) \, \times { 1}00\% $$

Afterward, the viability cells’ percentage was plotted versus the concentration of the anticancer agent. The IC_50_ value was calculated by fixing the viability cells’ percentage equals 50%. The in vitro anticancer assay for each sample was performed in three replications.

### Molecular docking study of hydroxyxanthone derivative as an anticancer agent

The human Topoisomerase II with 4G0V as the protein code was retrieved from the Protein Data Bank. At first, the water molecules were removed from the Topoisomerase II protein structure. Then, the hydrogen atoms were added to build up the structure of the Topoisomerase II protein using AutoDock Tools software. The molecular docking process was performed by using AutoDock Vina software. The validity of the molecular docking study is represented by the root-mean-square deviation (RMSD) value less than 2.0 Å^34^. Finally, the formed interactions between hydroxyxanthone and the active site of Topoisomerase II protein were visualized by using Discovery Studio software.

## Results and discussion

### Synthesis of hydroxyxanthone derivatives

**Figure 1:**
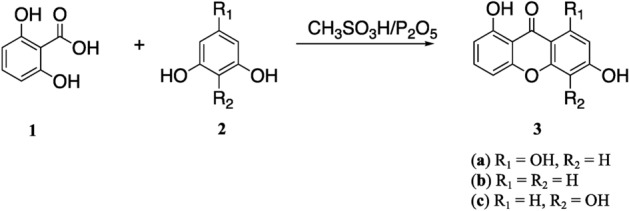
Synthesis scheme of hydroxyxanthone derivatives.

The synthesis scheme of hydroxyxanthone derivatives in this work is shown in Fig. 1. All hydroxyxanthones (**3a**–**c**) were prepared through a cyclo-acylation reaction of 2,6-dihydroxybenzoic acid (**1**) with phenolic derivatives (**2a**–**c**). From the spectroscopic elucidation, the correct chemical structure of hydroxyxanthones **3a**–**c** has been confirmed. The FTIR, MS, ^1^H–, and ^13^C–NMR spectra of hydroxyxanthones **3a**–**c** are depicted in Figs. S1–S12. In general, the FTIR spectra of hydroxyxanthones showed the appearance of O–H hydroxyl groups at 3425–3448 cm^−1^ while the C=O functional group appeared as a sharp signal at 1604–1612 cm^−1^. On the other hand, the C=C aromatic and C–O–C heterocyclic moieties were observed at 1418–1465 and 1273–1296 cm^−1^, respectively (Figs. S1, S5, and S9). Another evidence to prove the successful production of hydroxyxanthones was the presence of molecular ion fragments ([M]^+^) in their MS spectra (Figs. S2, S6, and S10). Hydroxyxanthones **3a** and **3c** showed five aromatic protons, while hydroxyxanthone **3b** showed six aromatic protons in the range of 6.09–7.99 ppm in their ^1^H–NMR spectra (Figs. S3, S7, and S11). Meanwhile, the presence of carbon atoms of the carbonyl group of hydroxyxanthones **3a**–**c** was found at 185.1, 181.9, and 182.3 ppm, respectively, in their ^13^C–NMR spectra (Figs. S4, S8, and S12). These spectroscopic data demonstrated that hydroxyxanthones had been successfully synthesized in this work.

### Antioxidant activity assay of hydroxyxanthones

**Figure 2:**
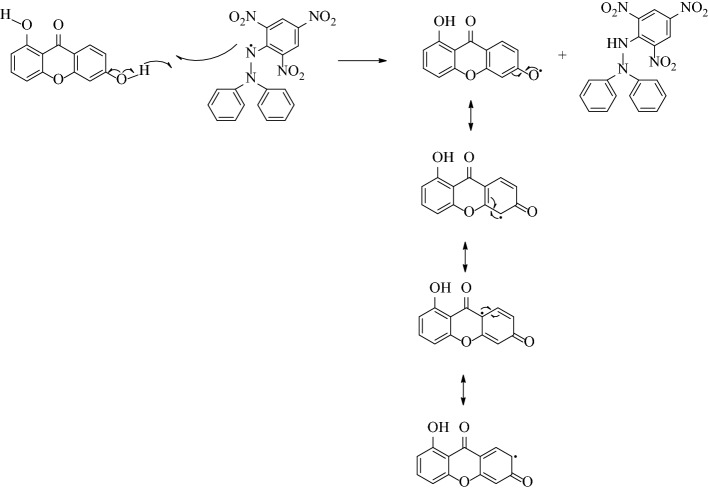
The plausible reaction of DPPH free radical with dihydroxyxanthone **3b**.

Antioxidant activity assay of hydroxyxanthones **3a**–**c** was performed by using DPPH free radicals method as the most common procedure. The antioxidant activity of hydroxyxanthones was shown in the term IC_50_ value (Table 1). A lower IC_50_ value means a higher antioxidant activity of hydroxyxanthones. Although all hydroxyxanthones gave a lower antioxidant activity than BHT as the positive control, the trihydroxyxanthones (compounds **3a** and **3c**) gave higher IC_50_ values than the dihydroxyxanthone **3b**. It shows that a higher amount of hydroxyl groups on the xanthone structure gave a lower antioxidant activity due to a stronger hydrogen bonding among the hydroxyl groups as previously reported^35,36^. Dihydroxyxanthone **3b** exhibited the strongest antioxidant activity (IC_50_ = 349 ± 68 µM). Since the hydroxyl groups were located far from each other, the DPPH free radicals could easily attack the hydroxyl groups of dihydroxyxanthone **3b**, yielding a strong antioxidant activity. This phenomenon was in agreement with the previous report^37^. The plausible reaction mechanism of DPPH free radicals with dihydroxyxanthone **3b** is shown in Fig. 2. This reaction mechanism generated a product with high resonance stability which could be responsible for the high antioxidant activity of dihydroxyxanthone **3b**.

**Table 1:** In vitro antioxidant activity of hydroxyxanthone derivatives.

Compound	IC_50_ (µM)
**3a**	653 ± 53
**3b**	349 ± 68
**3c**	524 ± 72
BHT	40 ± 4

### Anticancer activity assay of hydroxyxanthones

Evaluation of the anticancer activity of hydroxyxanthones was conducted through the MTT assay. The anticancer activity of hydroxyxanthones was represented in the term of IC_50_ value (see Table 2). A lower IC_50_ value reflects a stronger anticancer activity of hydroxyxanthones. Even though the anticancer activity of hydroxyxanthones was weaker than either doxorubicin or cisplatin as the positive control, the anticancer activity of hydroxyxanthones was still categorized as medium activity.

**Table 2:** In vitro anticancer activity of hydroxyxanthone derivatives.

Compound	IC_50_ (µM)			
	Vero^39^	MCF-7	WiDr	HeLa
**3a**	3395 ± 435	184 ± 15	254 ± 15	277 ± 9
**3b**	308 ± 35	450 ± 17	355 ± 24	322 ± 4
**3c**	224 ± 14	419 ± 27	209 ± 4	241 ± 13
Doxorubicin	150 ± 3	71 ± 6	3 ± 0.2	–
Cisplatin	–	–	–	142 ± 52

Among the prepared hydroxyxanthones, hydroxyxanthone **3a** was found as the best anticancer agent against the MCF-7 cell line (IC_50_ = 184 ± 15 µM) while hydroxyxanthone **3c** was the most active anticancer agent against WiDr (IC_50_ = 209 ± 4 µM) and HeLa (IC_50_ = 241 ± 13 µM) cell lines. These results demonstrated that trihydroxyxanthones were more active as the anticancer agent than dihydroxyxanthone **3b**. However, trihydroxyxanthone **3c** was more toxic to normal Vero cell lines (IC_50_ = 224 ± 14 µM) rather than either MCF-7 (IC_50_ = 419 ± 27 µM) or HeLa (IC_50_ = 241 ± 13 µM) cell lines. In contrast, trihydroxyxanthone **3a** was more toxic to MCF-7 (IC_50_ = 184 ± 15 µM), WiDr (IC_50_ = 254 ± 15 µM), and HeLa (IC_50_ = 277 ± 9 µM) cancer cell lines rather than normal Vero cell line (IC_50_ = 3395 ± 435 µM), which was remarkable.

Good anticancer drug candidates must be selective, i.e., toxic only to cancer cells but safe for normal cell lines^38^. The selectivity index (SI) was a critical parameter to describe selective anticancer activity. The SI value was calculated by dividing the IC_50_ of Vero cell lines with IC_50_ of cancer cells of hydroxyxanthone **3a**–**c**. A higher SI value describes a more selective anticancer agent. The SI value of hydroxyxanthones **3a**–**c** is listed in Table 3. Compound **3a** was found as the most selective anticancer agent due to its highest SI value against MCF-7, WiDr, and HeLa cancer cell lines among the other hydroxyxanthones in this work.

**Table 3:** Selectivity index of hydroxyxanthone derivatives as anticancer agent.

Compound	MCF-7	WiDr	HeLa
3a	18.42	13.39	12.25
3b	0.686	0.870	0.959
3c	0.535	1.072	0.931

Table 4 compares the anticancer activity of hydroxyxanthones **3a**–**c** and the previously reported hydroxyxanthones against WiDr cancer cell line. Compared to the other hydroxyxanthone derivatives, dihydroxyxanthones (IC_50_ = 355–1255 µM) gave higher IC_50_ values than trihydroxyxanthones (IC_50_ = 38–384 µM). Among the dihydroxyxanthones, the order of the anticancer activity against the WiDr cancer cell line was 1,6-dihydroxyxanthone > 3,6-dihydroxyxanthone > 1,3-dihydroxyxanthone > 3,4-dihydroxyxanthone. Meanwhile, the order of the anticancer activity of trihydroxyxanthones was 3,4,6-trihydroxyxanthone > 1,5,6-trihydroxyxanthone > 1,3,8-trihydroxyxanthone > 1,3,6-trihydroxyxanthone. From the reported quantitative structure–activity relationship (QSAR) study, we found that the IC_50_ value of hydroxyxanthone as the anticancer agent against WiDr cancer cell line depends on the net atomic charge on carbon atoms, dipole moment, and octanol/water partition coefficient^33^. Furthermore, the SI values of trihydroxyxanthones against WiDr cells (SI = 1.072–66.39) are higher than the dihydroxyxantones (SI = 0.870–2.230), demonstrating that trihydroxyxanthones are more potential anticancer drug candidates than dihydroxyxanthones.

**Table 4:** Comparison of in vitro anticancer activity of hydroxyxanthone derivatives against WiDr cancer cell line.

Compound	IC_50_ (µM)	SI	Reference
	WiDr		
1,3,8-trihydroxyxanthone (**3a**)	254 ± 15	13.39	This work
1,6-dihydroxyxanthone (**3b**)	355 ± 24	0.870	
1,5,6-trihydroxyxanthone (**3c**)	209 ± 4	1.072	
1,3-dihydroxyxanthone	836 ± 109	2.230	^33^
3,4-dihydroxyxanthone	1255 ± 105	1.160	
3,6-dihydroxyxanthone	786 ± 146	1.630	
1,3,6-trihydroxyxanthone	384 ± 93	1.540	
3,4,6-trihydroxyxanthone	38 ± 11	66.39	

### Molecular docking study on the inhibition of topoisomerase II protein by hydroxyxanthones

The experimental in vitro assay found that hydroxyxanthone **3a** exhibited the strongest and the most selective anticancer activity among the other hydroxyxanthones in this work. Therefore, a further investigation was conducted for hydroxyxanthone **3a** through a molecular docking study to reveal the inhibition mechanism on the active site of Topoisomerase II protein. Topoisomerase II protein is selected as the target protein since Topoisomerase II serves a pivotal role in the DNA replication and transcription of cancer cells. Doxorubicin is an anticancer agent with strong inhibitory activity towards Topoisomerase II protein. Doxorubicin consists of two fused aromatics with a carbonyl structure, which is similar to the prepared hydroxyxanthone **3a**.

**Figure 3:**
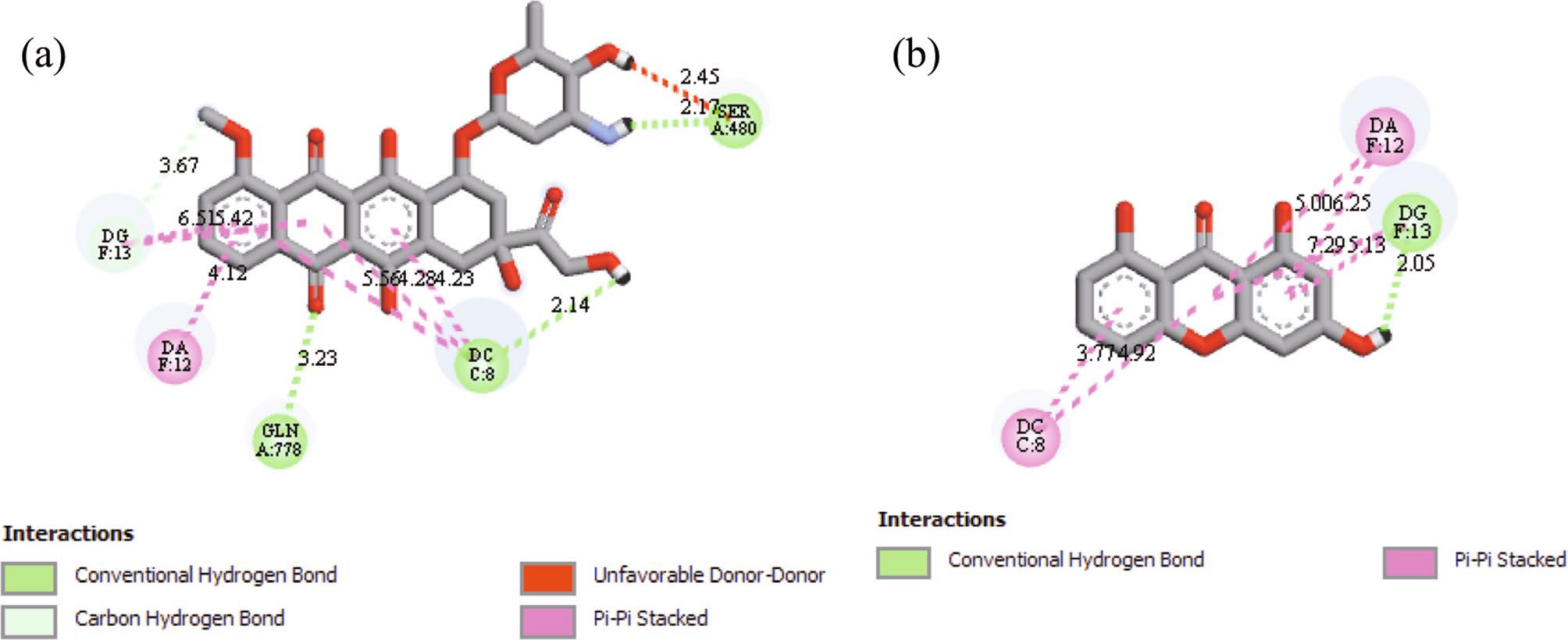
Visualization of the intermolecular interactions of (**a**) doxorubicin and (**b**) hydroxyxanthone **3a** with the active sites of topoisomerase II.

The formed interactions from the molecular docking study are displayed in Fig. 3. Several interactions were observed between hydroxyxanthone **3a** with the DNA chains of Topoisomerase II, such as hydrogen bond with DG13, as well as π–π stacking with DA12 and DC8. These kinds of interactions have been reported to be critical for the inhibitory activity against Topoisomerase II protein. Etoposide, a standard Topoisomerase inhibitor, was reported to generate hydrogen bond interactions with Asp463 and DG13 on the active site of Topoisomerase II^40^. On the other hand, the binding interaction of the anticancer agent with DG13 was reported as pivotal interaction as this interaction stimulates the formation of DNA damage that is toxic to cancer cells^41^. Similar binding modes with DA12 and DC8 through π–π stacking were also reported for the most active benzoxazole and olivacine derivatives as the anticancer agents^40,42^.

On the other side, doxorubicin interacted to the active site of Topoisomerase II through hydrogen bond (Ser480, DC8, Gln778), carbon-hydrogen bond (DG13), and π–π stacking (DG13, DA12, DC8) interactions. Compared to doxorubicin, hydroxyxanthone **3a** generated no interaction with Ser480 and Gln778. Therefore, the theoretical binding energy and binding constant of hydroxyxanthone **3a** were lower than doxorubicin. The binding energy and binding constant of hydroxyxanthone **3a** with Topoisomerase II were −8.0 kcal mol^−1^ and 1.362 µM, respectively, which were weaker than doxorubicin (−8.9 kcal mol^−1^, 0.299 µM). Nevertheless, it is worthy to note that hydroxyxanthone **3a** was able to disturb the function of Topoisomerase II protein, thus increasing the cell apoptosis, which led to the death of cell cancers as previously reported^31^.

## Conclusions

Three hydroxyxanthones (**3a**–**c**) have been successfully synthesized from 2,6-dihydroxybenzoic acid and phenolic compounds through an acylation reaction using Eaton’s reagent. The chemical structures of hydroxyxanthones have been clearly elucidated by using FTIR, MS, ^1^H–, and ^13^C–NMR. The in vitro antioxidant assay showed that trihydroxyxanthones **3a** and **3c** gave higher IC_50_ values than dihydroxyxanthone **3b** due to stronger hydrogen bonds. It was found that dihydroxyxanthone **3b** exhibited the strongest antioxidant activity (IC_50_ = 349 ± 68 µM). On the other hand, trihydroxyxanthones gave higher anticancer activity than the dihydroxyxanthone one. Trihydroxyxanthone **3a** exhibited higher toxicity against MCF-7 (IC_50_ = 184 ± 15 µM), WiDr (IC_50_ = 254 ± 15 µM), and HeLa (IC_50_ = 277 ± 9 µM) cancer cell lines rather than normal Vero cells (IC_50_ = 3395 ± 435 µM). Consequently, trihydroxyxanthone **3a** yielded the highest selectivity index (12.25–18.42) among the prepared hydroxyxanthones in this work. The molecular docking study reveals that hydrogen bond and π–π stacking interactions were observed between trihydroxyxanthone **3a** with the DNA chains of Topoisomerase II. The trihydroxyxanthone **3a** was able to disturb the function of Topoisomerase II protein. These findings are important for the future development of anticancer agents based on hydroxyxanthone derivatives.

## Supplementary Information

Supplementary Figures.

## Data Availability

The datasets generated during and/or analyzed in the current study are available from the corresponding author upon a reasonable request.

## References

[1] Liguori I (2018). Oxidative stress, aging, and diseases. Clin. Interv. Aging.

[2] Gutteridge JMC, Halliwell B (2018). Mini-review: Oxidative stress, redox stress or redox success?. Biochem. Biophys. Res. Commun..

[3] Phaniendra A, Jestadi DB, Periyasamy L (2015). Free radicals: Properties, sources, targets, and their implication in various diseases. Indian J. Clin. Biochem..

[4] Estenbauer H, Rothemeder MD, Waeg G (1991). Role of vitamin E in preventing the oxidant of low density lipoprotein. Am. J. Clin. Nutr..

[5] Neha K, Haider MR, Pathak A, Yar MS (2019). Medicinal prospects of antioxidants: A review. Eur. J. Med. Chem..

[6] Gulcin I (2020). Antioxidants and antioxidant methods: An updated overview. Arch. Toxicol..

[7] Sindhi V (2013). Potential applications of antioxidants - A review. J. Pharm. Res..

[8] Lourenco SC, Moldao-Martins M, Alves VD (2019). Antioxidants of natural plant origins: From sources to food industry applications. Molecules.

[9] Kanwal K (2021). Indole-3-acetamides: As potential antihyperglycemic and antioxidant agents; synthesis, in vitro α-amylase inhibitory activity, structure-activity relationship, and in silico studies. ACS Omega.

[10] Qureshi, F., et al. Synthesis and characterization of cadmium-bismuth microspheres for the catalytic and photocatalytic degradation of organic pollutants, with antibacterial, antioxidant and cytotoxicty assay. *J. Photochem. Photobiol. B Biol.* **202**, 111723. 10.1016/j.jphotobiol.2019.111723 (2020). 10.1016/j.jphotobiol.2019.111723 31785448

[11] Rafique R (2020). Synthesis, in vitro α-amylase inhibitory, and radicals (DPPH & ABTS) scavenging potentials of new *N*-sulfonohydrazide substituted indazoles. Bioorg. Chem..

[12] Jamil W (2019). Syntheses, characterization, in vitro antiglycation and DPPH radical scavenging activities of isation salicylhydrazidehydrazone and its Mn(II), Co(II), Ni(II), Cu(II), and Zn(II) metal complexes. Arab. J. Chem..

[13] Silva V (2019). 1,2-Dihydroxyxanthone: Effect on A375–C5 melanoma cell growth associated with interference with THP-1 human macrophage activity. Pharmaceuticals.

[14] Castanheiro RAP, Silva AMS, Campos NAN, Nascimento MSJ, Pinto MMM (2009). Antitumor activity of some prenylated xanthones. Pharmaceuticals.

[15] Lippold T, Neudorfl JM, Griesbeck A (2021). New acridone- and (thio)xanthone-derived 1,1-donor-acceptor-substituted alkenes: pH-Dependent fluorescence and unusual photooxygenation properties. Molecules.

[16] Kurniawan YS (2021). An update on the anticancer activity of xanthone derivatives: A review. Pharmaceuticals.

[17] Gunter NV, The SS, Lim YM, Mah SH (2020). Natural xanthones and skin inflammatory diseases: Multitargeting mechanisms of action and potential application. Front. Pharmacol..

[18] Shagufta, Ahmad I (2016). Recent insight into the biological activities of synthetic xanthone derivatives. Eur. J. Med. Chem..

[19] Zhang H (2020). Anticancer activity of dietary xanthone α-mangostin against hepatocellular carcinoma by inhibition of STAT3 signaling via stabilization of SHP1. Cell Death Dis..

[20] Pinto MM (2021). From natural products to new synthetic small molecules: A journey through the world of xanthones. Molecules.

[21] Franca F (2020). A pyranoxanthone as a potent antimitotic and sensitizer of cancer cells to low doses of paclitaxel. Molecules.

[22] Nkengfack EA, Kounga PA, Fomun TZ, Meyer M, Bodo B (2002). Glubixanthones A and B, two new cytotoxic xanthones with isoprenoid groups from the root bark of *Symphonia globurifer*. J. Nat. Prod..

[23] Zarena AS, Sankar KU (2009). Supercritical carbon dioxide extraction of xanthones with antioxidant activity from *Garcinia mangostana*: Characterization by HPLC/LC-ESI-MS. J. Supercrit. Fluids.

[24] Loureiro DRP (2019). Structures, activities and drug-likeness of anti-infective xanthone derivatives isolated from the marine environment: A review. Molecules.

[25] Sousa ME, Pinto MMM (2005). Synthesis of xanthones: An overview. Curr. Med. Chem..

[26] Zhou BD (2018). Synthesis and antitumor, antityrosinase, and antioxidant activities of xanthone. J. Asian Nat. Prod. Res..

[27] Taha M (2019). Synthesis, anticancer, molecular docking and QSAR studies of benzylhydrazone. J. Saudi Chem. Soc..

[28] Abbass EM, Khalil AK, Mohamed MM, Eissa IH, El-Naggar AM (2020). Design, efficient synthesis, docking studies, and anticancer evaluation of new quinoxalines as potential intercalative topo II inhibitors and apoptosis inducers. Bioorg. Chem..

[29] Ito C (2003). Chemical constituents of *Garcinia fusca*: Structure elucidation of eight new xanthones and their cancer chemopreventive activity. J. Nat. Prod..

[30] Kuete V (2014). Cytotoxicity and modes of action of three naturally occuring xanthones (8-hydroxycudraxanthone G, murosignin I and cudraxanthone I) against sensitive and multi-drug-resistant cancer cell lines. Phytomedicine.

[31] Jun KY (2011). Synthesis, biological evaluation, and molecular docking study of 3-(3′-heteroatom substituted-2′-hydroxy-1′-propyloxy) xanthone analogues as novel topoisomerase IIα catalytic inhibitor. Eur. J. Med. Chem..

[32] Graves DE, Velea LM (2000). Intercalative binding of small molecules to nucleic acids. Curr. Org. Chem..

[33] Miladiyah I, Jumina J, Haryana SM, Mustofa M (2018). Biological activity, quantitative structure-activity relationship analysis, and molecular docking of xanthone derivatives as anticancer drugs. Drug Des. Dev. Ther..

[34] Trott O, Olson AJ (2010). AutoDock Vina: Improving the speed and accuracy of docking with new scoring function, efficient optimization and multithreading. J. Comput. Chem..

[35] Freitas VLS, da Silva MDMCR (2018). Influence of hydroxyl functional group on the structure and stability of xanthone: A computational approach. Molecules.

[36] Jumina J (2019). Development of C-arylcalix[4]resorcinarenes and C-arylcalix[4]pyrogallolarenes as antioxidant and UV-B protector. Indones. J. Chem..

[37] Franca HS, Rocha L, Fernande CP, Ruis ALT, Carvalho JE (2013). Antiproliferative activity of the hexanic extract and phloroglucinols from *Hypericum brasiliense*. Rev. Bras. Pharmacogn..

[38] Khan T (2020). Anticancer plants: A review of the active phytochemicals, applications in animal models, and regulatory aspects. Biomolecules.

[39] Zakiah M (2021). *In vitro* antiplasmodial, heme polymerization, and cytotoxicity of hydroxyxanthone derivatives. J. Trop. Med..

[40] Tylinska B (2021). Evaluation of interactions of selected olivacine derivatives with DNA and topoisomerase II. Int. J. Mol. Sci..

[41] Lemke K (2005). Induction of unique structural changes in guanine-rich DNA regions by the triazoloacridone C-13, a topoisomerase II inibitor with antitumor activities. Nucleic Acids Res..

[42] Oksuzoglu E (2016). Antitumor activities on HL-60 human leukemia cell line, molecular docking, and quantum-chemical calculations of some sulfonamide-benzoxazoles. Artif. Cells Nanomed. Biotechnol..

