# Supplementary material for: Synthesis and in vitro assay of hydroxyxanthones as antioxidant and anticancer agents
Source: Sci Rep. 2022 Jan 27;12:1535. doi: 10.1038/s41598-022-05573-5 (PMC8795354; doi:10.1038/s41598-022-05573-5)
Supplement: Supplementary file 1 — Supplementary Figures. [file 41598_2022_5573_MOESM1_ESM.docx]

Supplementary Information

**Synthesis and *In Vitro* Assay of Hydroxyxanthones As Antioxidant and Anticancer Agents**

Nela Fatmasari^1^, Yehezkiel Steven Kurniawan^1^, Jumina^1,^*, Chairil Anwar^1^,

Yoga Priastomo^1^, Harno Dwi Pranowo^1^, Abdul Karim Zulkarnain^2^ and

Eti Nurwening Sholikhah^3^

^1^Department of Chemistry, Faculty of Mathematics and Natural Science, Universitas Gadjah Mada, Yogyakarta 55281, Indonesia

^2^Department of Pharmaceutical Technology, Faculty of Pharmacy, Universitas Gadjah Mada, Yogyakarta 55281, Indonesia

^3^Department of Pharmacology and Therapy, Faculty of Medicine, Public Health, and Nursing, Universitas Gadjah Mada, Yogyakarta 55281, Indonesia

*Corresponding author: [jumina@ugm.ac.id](mailto:jumina@ugm.ac.id) Tel.: +62 274 545188

Figure S1. FTIR spectrum of compound **3a**

Figure S2. MS spectrum of compound **3a**

Figure S3. ^1^H-NMR spectrum of compound **3a**

Figure S4. ^13^C-NMR spectrum of compound **3a**

Figure S5. FTIR spectrum of compound **3b**

Figure S6. MS spectrum of compound **3b**

Figure S7. ^1^H-NMR spectrum of compound **3b**

Figure S8. ^13^C-NMR spectrum of compound **3b**

Figure S9. FTIR spectrum of compound **3c**

Figure S10. MS spectrum of compound **3c**

Figure S11. ^1^H-NMR spectrum of compound **3c**

Figure S12. ^13^C-NMR spectrum of compound **3c**
